# The onset of sleep disturbances and their associations with anxiety after acute high-altitude exposure at 3700 m

**DOI:** 10.1038/s41398-019-0510-x

**Published:** 2019-07-22

**Authors:** Shi-Zhu Bian, Laiping Zhang, Jun Jin, Ji-Hang Zhang, Qian-Ning Li, Jie Yu, Jian-Fei Chen, Shi-Yong Yu, Xiao-Hui Zhao, Jun Qin, Lan Huang

**Affiliations:** 1 Institute of Cardiovascular Diseases, Xinqiao Hospital, Army Medical University (Third Military Medical University), Chongqing, China; 2 Department of Cardiology, Xinqiao Hospital, Army Medical University (Third Military Medical University), Chongqing, China; 3 Department of Neurology, Xinqiao Hospital, Army Medical University (Third Military Medical University), Chongqing, China

**Keywords:** Human behaviour, Psychiatric disorders

## Abstract

Sleep disturbances and psychiatric repercussions pose great challenges at high altitude; however, few studies have investigated sleep disturbance and anxiety profiles and their associations after acute exposure in consecutive patients. Thus, we aimed to study the profiles of sleep disturbances in consecutive patients after high-altitude exposure and the association of such disturbances with anxiety. A total of 668 participants were recruited at sea level and 3700 m. The trials were performed at sea level (1 week prior to a 2-h flight to a high-altitude destination) and at 3700 m (24, 72, and 168 h). Sleep disturbances were assessed by self-reported sleep patterns and scores on the Athens Insomnia Scale (AIS). State anxiety was assessed using the Self-Rating Anxiety Scale (SAS). In our study, the incidence of sleep disturbances increased significantly after acute high-altitude exposure (65.3%, 434/668) and then gradually decreased after 72 h (50%, 141/282) and 168 h (44%, 124/282). The sleep assessments AIS [2.0 (4.0) vs. 4.0 (5.0)] and ESS [4.0 (4.0) vs. 5.0 (5.0)] increased significantly (*p* < 0.05). Also, the SAS increased significantly from 26.25 (3.75) to 28.75 (7.5). The SAS was significantly high in sleep disturbance group [31.25 (7.5) vs. 27.5 (5), *p* < 0.001] than in the non-sleep- disturbance group. The baseline SAS and AIS scores were significantly higher in participants with sleep disturbances than in those without (*p* < 0.01). Age, baseline insomnia, sleepiness, fatigue, and higher SAS were predictors of sleep disturbances in univariate regression (all *p* values < 0.05). However, only an older age (*p* = 0.045) and a higher baseline SAS (*p* = 0.018) remained independent predictors of sleep disturbances. Our findings indicated that acute high-altitude exposure triggers the onset of sleep disturbances, which are closely associated with anxiety. Furthermore, baseline state anxiety and age are independent predictors of sleep disturbances at high altitude.

## Introduction

Sleep architecture and continuity have been demonstrated to be impaired after high-altitude exposure, even after a short time-stay at high altitude^1^. The majority of individuals (32–74%) suffer from extremely poor sleep quality or insomnia at high altitude, particularly during the first few days^2–4^; this poor sleep quality reduces day-time cognitive performance, induces general malaise and contributes to the development of acute mountain sickness (AMS)^5,6^. Sleep disturbances, usually including insomnia, impaired sleep continuity and daytime drowsiness, are the most common and primary complaints after high-altitude stress^7^. Hypobaric hypoxia-related sleep alterations, which are also termed sleep disturbances, have been demonstrated to adversely affect daytime performance or induce drowsiness^8^.

Insomnia has been used as a symptom of AMS in the Lake Louise scoring system^9^, although it has been removed from the diagnosis criteria in the new version of the scoring system (2018 version)^10^. The destruction of sleep structure and arousals from sleep in lowlanders, measured by objective or subjective measurements, have also been observed previously. Though most symptoms of AMS disappear after 2 or 3 days; however, the sleep disturbance may persist. Previous studies have assessed sleep disturbances by using Groningen Sleep Quality Questionnaire (score >6)^11^ and polysomnography indicating that high-altitude induces sleep disorders^12^. Though, it has been separated from AMS score system, the insomnia is still the one of the most frequent symptom at high altitude. In addition, though the symptoms of AMS disappear after 2 or 3 days;^12^ however, the sleep disturbances may persist. Thus, the high-altitude induced sleep disturbances have been studied separately.

Sleep quality and sleep disturbances have also been shown to be affected by emotional and psychiatric conditions such as anxiety and depression at sea level^13^. High-altitude exposure also induces anxiety or aggravates state anxiety^14,15^. Furthermore, negative mood status and psychiatric status as well as poor sleep quality and insomnia have also been widely documented by our research and that of others^7,13^.

Although studies that focus on sleep and anxiety at sea level have been performed, the precise associations, including causal associations, between high-altitude-related sleep disturbances, and state anxiety after acute high-altitude exposure have not been fully uncovered^3,11,16^. Furthermore, two of our previous studies have indicated that the increased anxiety at high altitude by using Symptom Checklist-90 and mood status profile examinations^14,17^. However, these others’ studies on high-altitude induced sleep disturbances either performed in a small sample size or in a relative slow ascent rate (ascent times varied from several days to 35 days; altitude varied from 1400 to 5000 m) or in the stimulated hypoxic chamber^4,12^. The high-altitude sleep disturbances may not so typical with less incidence or severity. Furthermore, there were only few studies focusing on associations between anxiety and high-altitude sleep disturbances. In addition, the roles of state anxiety in high-altitude induced sleep disturbances are still in debate.

Based on previous theoretical and practical investigations as well as their limitations, we hypothesized that high-altitude induced sleep disturbances are closely associated with state anxiety and performed this field trial in a large sample size of populations after acute high-altitude exposure at 3700 m in a fast (within 2 h flight) transport pattern to consecutively observe the onset of altitude-induced sleep disturbances by using three dimensions of scores and to identify their associations with anxiety after acute hypoxia.

## Methods

### Participants

The participants (*n* = 668) in this study were recruited according to the inclusion and exclusion criteria in June and July of 2012 in Chengdu, Sichuan province (sea level, average 500 m) and Lhasa (3700 m above sea level). Only healthy males between 18 and 40 years of age were included. Subjects with any of the following diseases were excluded: sleep apnea syndrome, hypertension, arrhythmia, myocarditis or other cardiovascular disease, primary headache, cold, pneumonia, pulmonary tuberculosis or other respiratory disease, disorders of the liver or kidneys, malignant tumors, and neuropsychosis.

This study was reviewed and approved by the Ethics Committee of Xinqiao Hospital, Army Medical University. The purpose and procedures of the study were thoroughly described to all subjects who agreed to participate, and all of the participants signed informed consent forms before their examinations.

### Procedures and clinical data collection

Our field trial for the baseline data collection was performed within 1 week prior to the flight (a 2-h plane ride that transported the participants to a high-altitude destination on 29 June or 1 July 2012). The trials at high altitude were performed within 18 to 24 h, at 72 h (subgroup, *n* = 282) and at 168 h (subgroup) after arrival at 3700 m. The trials of this study are shown in Supplementary Fig. S1 (Supplementary Material 1).

Demographic data [i.e., age, body mass index (BMI), smoking history, and alcohol consumption] were collected using a structured case-report form (CRF, Supplementary Material 2 a representative CRF). Sleep quality was assessed by self-reported sleep patterns (0 = slept as well as usual; 1 = slept poorer than before; 2 = woke up several times; 3 = could not fall asleep last night) and Athens Insomnia Scale (AIS) insomnia scores (Supplementary Material 3 AIS score test). Sleepiness was also assessed through self-report (0 = without sleepiness; 1 = with sleepiness) and the Epworth Sleepiness Scale (ESS, Supplementary Material 4 ESS score test). Effects on activity were reported as follows: 0 = no activity reduction; 1 = mild activity reduction; 2 = moderate activity reduction; and 3 = severe activity reduction. Fatigue was also classified from 0 to 3 depending on the extent of fatigue. Further detailed assessments were measured using a quantitative score (Fatigue Self-Assessment Scale, FSAS, Supplementary Material 5 FSAS test). Anxiety was assessed using the widely employed Self-Rating Anxiety Scale (SAS, Supplementary Material 6 SAS test) in Chinese. Systolic blood pressure (SBP), diastolic blood pressure (DBP), heart rate (HR), and pulse oxygen saturation (SpO_2_) were also measured at baseline and at 3700 m after the participants rested for 30 min in a sitting position.

### Definitions of variables

Sleep disturbances at high altitude were defined as difficulty falling asleep, waking several times or poorer-than-usual sleep quality, or an AIS score >6. In the present study, the term “sleep disturbances” refers to self-reported poor sleep quality, clinical insomnia, and/or alterations or deficits in sleep parameters.

Smoking status was defined as smoking 1 or more cigarettes per day for at least 1 year. Alcohol users were defined as those who drank once a week (clear spirits, beer, or red wine).

### Statistical analysis

**Fig. 1:**
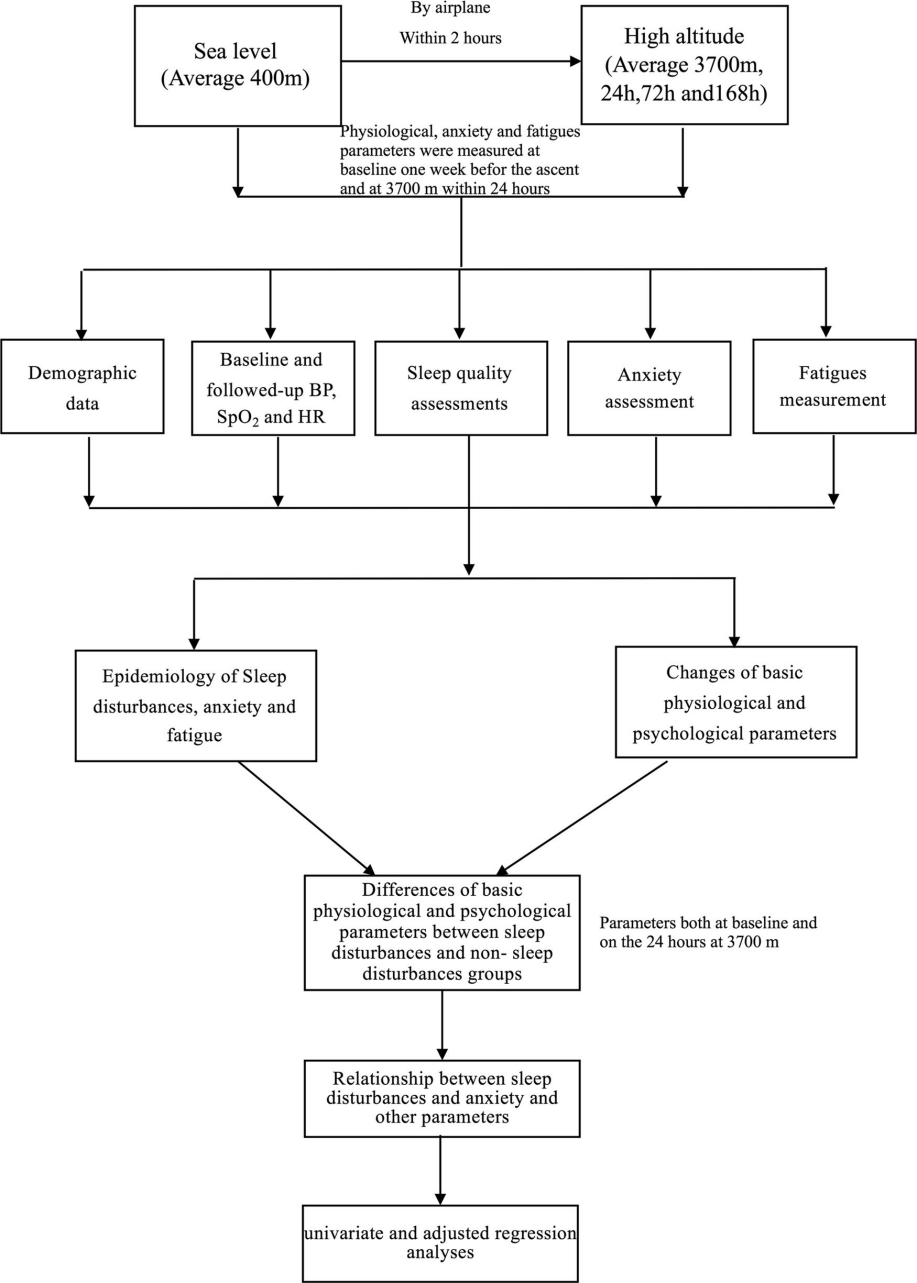
**The flow chart of our study and its statistical analyses**

Normally distributed variables, including age, BMI, HR, SBP, DBP, and SpO_2_, were expressed as the mean ± SD and were compared using independent sample *T* tests. Non-normally distributed variables, such as the SAS, AIS, ESS, and FSAS scores (including their changes from baseline to high altitude), were expressed as median (interquartile) and compared with nonparametric tests. Furthermore, categorical and noncontinuous variables were presented as percentages (cases) and were compared using the chi-squared test. The associations between AIS score and other parameters were analyzed by Pearson’s correlation. Univariate and adjusted logistic regressions were used to identify predictors and risk factors for sleep disturbances. A flow chart of the analysis process is provided in Fig. 1. *p* ≤ 0.05 was considered statistically significant. The statistical analyses were performed in SPSS 19.0 for Windows. All statistical methods and results were reviewed and approved by statisticians from the Army Military Medical University. The details of the study process were shown in Supplementary Material 7 Detailed Methods.

## Results

### Demographic data and history

The mean age and BMI of the subjects in the study were 22.88 ± 3.77 years and 21.04 ± 2.06 kg/m^2^, respectively. Altogether, 55.2% and 4.8% of the participants smoked and drank alcohol, respectively.

### Profiles of sleep quality, anxiety, and fatigue

**Fig. 2:**
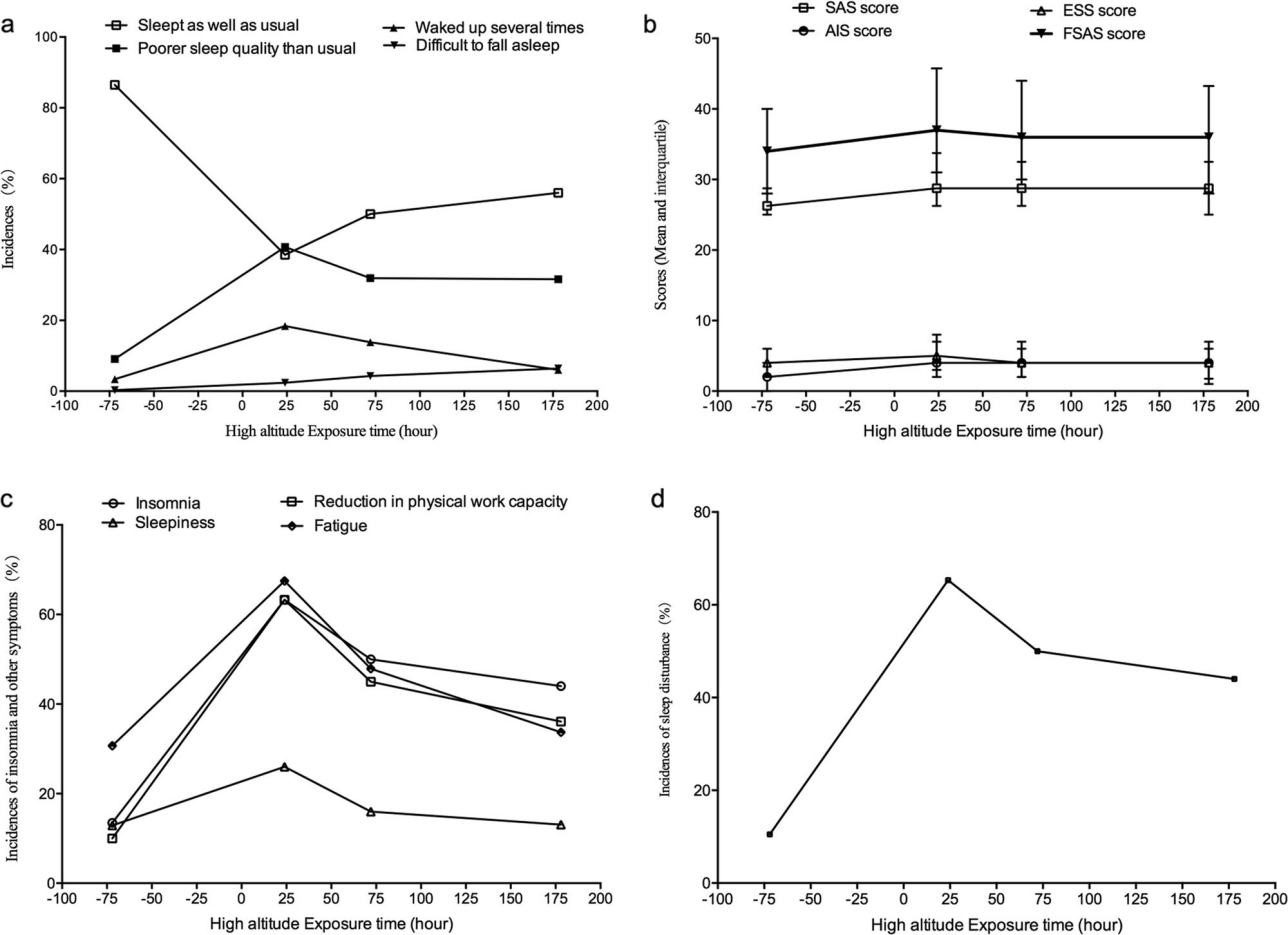
The incidence rates of insomnia, fatigue, and sleepiness. **a** The incidence of various types of insomnia increased significantly after acute high-altitude exposure and decreased with exposure time. **b** The changes in SAS, AIS, ESS and FSAS scores: increased significantly after acute high-altitude exposure then decreased gradually. **c** The incidences of insomnia, sleepiness and reduction in physical work capacity. **d** The incidences of sleep disturbances at different cross-sections

After exposure to 3700 m, the incidences of insomnia and sleepiness significantly increased (Fig. 2a, b), while they gradually recovered as the duration of exposure increased; however, they remained higher than baseline values. The AIS and ESS scores also increased upon acute high-altitude exposure and then decreased with the stay duration. In our study the incidence of sleep disturbances increased significantly after acute high altitude exposure (65.3%, 434/668) and then gradually decreased after 72 h (50%, 141/282) and 168 h (44%, 124/282).

Individuals’ SAS scores also significantly increased within 24 h at high altitude. Then, they decreased to a level that was higher than baseline at 72 and 168 h. In addition, the incidences of fatigue and reduction in physical work showed a pattern similar to that for insomnia (Fig. 2c), and the FSAS score results were similar to the SAS, ESS, and AIS score results (Fig. 2b). Incidence of sleep disturbances has also been shown in Fig. 2d indicating that it significantly raised accompanied a decrease after stay at high altitude.

### Differences in parameters between the sleep-disturbance and non-sleep-disturbance groups

Participants in the sleep-disturbance group were significantly older than those in the non-sleep-disturbance group (*p* = 0.011). Furthermore, at baseline, the incidence rates of insomnia (*p* = 0.006), sleepiness (*p* = 0.026), and fatigue (*p* < 0.001) were higher in the sleep-disturbance group. The incidence of reduction in physical work capacity (*p* = 0.022) was also elevated (Table 1).

**Table 1:** The variables between the sleep disturbances and nongroups after acute high-altitude exposure

	Sleep disturbances− (*n* = 234)	Sleep disturbances+ (*n* = 434)	*p*
*Demographic data*			
Age	22.38 ± 3.65	23.15 ± 3.80	0.011^*^
BMI	21.70 ± 2.09	21.76 ± 2.06	0.750
Current smoking	58.1% (136)	53.7% (233)	0.228
Current drinking	5.1% (12)	4.6% (20)	0.540
History of high-altitude exposure	26.9% (63)	30.0% (130)	0.410
*Parameters at sea level*			
Insomnia (n)	8.5% (20)	16.1% (70)	0.006^**^
Sleepiness (n)	9.0% (21)	15.0% (65)	0.026^*^
Fatigues (n)	20.5% (48)	36.2% (157)	<0.001^**^
Reduction in physical work capacity	6.4% (15)	12.0% (50)	0.022^*^
SAS	25 (2.5)	26.25 (3.75)	<0.001^**^
ESS	3 (4)	4 (4)	0.001^**^
AIS	2 (3)	2.5 (4)	<0.001^**^
FSAS	33 (11.25)	35 (13)	<0.001^**^
SBP	114.80 ± 11.84	115.74 ± 10.51	0.291
DBP	72.68 ± 9.04	73.57 ± 9.51	0.242
HR	66.50 ± 10.76	65.76 ± 9.90	0.375
SpO_2_	98.17 ± 0.95	98.10 ± 1.04	0.397
*At 3700* *m after acute exposure*			
Sleepiness	15.0% (35)	32.0% (139)	<0.001^**^
Fatigues	44.4% (104)	80% (347)	<0.001^**^
Reduction in physical work capacity	41.9% (98)	74.9% (225)	<0.001^**^
SAS	27.5 (5)	31.25 (7.5)	<0.001^**^
AIS	2 (4)	6 (4)	<0.001^**^
ESS	4 (4)	6 (6)	<0.001^**^
FSAS	34 (11)	40 (15)	<0.001^**^
SBP	118.88 ± 11.05	119.19 ± 12.08	0.744
DBP	78.75 ± 10.33	79.04 ± 9.92	0.721
HR	83.23 ± 11.57	85.08 ± 12.53	0.062
SpO_2_	89.13 ± 3.01	88.75 ± 3.14	0.375
*Changes from sea level to high altitude*			
SAS	1.25 (3.75)	3.75 (6.25)	<0.001^**^
ESS	1 (3)	2 (4)	<0.001^**^
AIS	0 (3)	3 (2)	<0.001^**^
FSAS	2 (8)	5 (13)	<0.001^**^
SBP	3 (16)	4 (16)	0.647
DBP	6 (15)	6 (15)	0.453
HR	16.76 ± 13.17	19.32 ± 12.54	0.013^*^
SpO_2_	−9.04 ± 3.07	−9.35 ± 3.26	0.239

The sleep disturbances populations were characterized by higher age, SAS, ESS, and FSAS score as well as HR

*SAS* Self-Rating Anxiety Scale, *ESS* Epworth Sleepiness Scale, *FSAS* Fatigue Self-Assessment Scale, *AIS* Athens Insomnia Scale, *SBP* Systolic blood pressure, *DBP* diastolic blood pressure, *HR* heart rate, *SpO*_*2*_ pulse oxygen saturation, *BMI* body mass index

*p < 0.05; **p < 0.01

Regarding sleepiness, anxiety, insomnia and fatigue, SAS, AIS, ESS, and FSAS scores were significantly higher in the sleep-disturbance population than in the non-sleep-disturbance population (all *p* values < 0.01).

However, smoking and drinking status as well as history of exposure to high altitude did not significantly differ between the sleep-disturbance and non-sleep-disturbance groups (all *p* > 0.05, Table 1). Furthermore, the baseline values of SBP, DBP, HR, and SpO_2_ were similar in the two groups. In the first 24 h, the differences in anxiety, sleepiness, fatigue and activity reduction, and their scores showed a similar pattern to the values observed at baselines (all *p* values < 0.05). A significant difference was observed for the change in SAS, ESS, AIS, and FSAS (all *p* values < 0.05) scores as well as for the change in HR (*p* = 0.013).

### Associations of sleep disturbances with SAS scores and other parameters

The AIS score after acute high-altitude exposure was closely related to the baseline severity of fatigue and to the reduction in physical work capacity (Supplementary Material 8 and Supplementary Table S1). We also found that the AIS score was closely associated with the SAS, ESS and FSAS scores (all *p* values < 0.001). At 3700 m, the AIS score was also significantly associated with fatigue severity, the reduction in activity capacity, and the SAS, ESS, and FSAS scores (all *p* values < 0.001). In addition, HR was positively associated with the AIS score, while SpO_2_ was significantly negatively correlated with the AIS score (Supplementary Material 8 and Supplementary Table S2).

Regarding changes in these parameters, excluding the SBP and DBP, the changes in the other parameters were significantly associated with the AIS score reported on the first day at 3700 m.

### Sleep disturbances and fatigue among the SAS alteration groups

A few individuals’ SAS scores decreased or remained unchanged, although the average SAS score increased from baseline to high altitude. Thus, we analyzed sleep disturbances among the groups with increased, unchanged, and decreased SAS. The baseline incidences of insomnia, fatigue, sleepiness, reduction in physical work capacity, and the AIS and ESS scores were lowest in the unchanged-SAS group (Table 2). However, the baseline values for insomnia, fatigue, sleepiness, and reduction in physical work capacity as well as the AIS and ESS scores were higher in the SAS-decreased group than in the increased-SAS group (Table 2). At 3700 m, the baseline values for insomnia, fatigue, sleepiness, and reduction in physical work capacity as well as the AIS and ESS scores were higher in the increased-SAS group than in the decreased-SAS group.

**Table 2:** Comparisons of insomnia among groups divided by change of SAS

Changes of SAS	Decreased (*n* = 83)	Unchanged (*n* = 122)	Increased (*n* = 463)	*p*
*Baseline parameters*				
Age	23.19 ± 3.49	23.20 ± 3.50	22.74 ± 3.88	0.352
BMI	21.51 ± 1.85	21.89 ± 2.08	21.74 ± 2.1	0.429
Insomnia	31.3% (26)	7.4% (9)	11.9% (55)	<0.001^**^
Sleepiness	24.1% (20)	9.8% (12)	11.7% (54)	0.004^**^
ESS	5 (4)	3 (5)	4 (4)	<0.001^**^
AIS	5 (4)	1 (3)	2 (4)	<0.001^**^
*Parameters at 3700* *m*				
Insomnia	55.4% (46)	38.5% (47)	68.7% (318)	<0.001^**^
Sleepiness	22% (19)	15.6 (19)	29.4% (136)	0.007^**^
Sleep disturbances	60.2% (50)	39.3% (48)	72.6% (336)	<0.001^**^
ESS	5 (3)	3 (5)	5 (5)	<0.001^**^
AIS	4 (6)	2 (4)	5 (5)	<0.001^**^

The SAS unchanged group showed lowest incidences of insomnia, sleepiness, and sleep disturbances

**p < 0.01

### Regression analysis and predictors of sleep disturbances

Age and baseline insomnia, sleepiness, fatigue, and reduction in physical work capacity as well as SAS, ESS, AIS, and FSAS scores were significantly associated with sleep disturbances. Furthermore, the follow-up sleepiness, fatigue, reduction in activity capacity, SAS, ESS and FSAS scores were also associated with sleep disturbances. The changes in SAS, ESS, FSAS, and AIS scores as well as HR also showed close associations with sleep disturbances (Supplementary Material 8 and Supplementary Table S2).

In the adjusted regression, the combination of older age and a higher baseline SAS score was found to increase the risk for sleep disturbances. For the parameters at 3700 m, in addition to age, the presence of a reduction in physical work capacity and fatigue as well as higher SAS and ESS scores were also risk factors for sleep disturbances. In addition, changes in HR and SAS score were associated with the risk of sleep disturbances, as was age (Table 3).

**Table 3:** Adjusted logistic regression for sleep disturbances

	β	OR	95%CI	95%CI	*p*
*Parameters at sea level*					
Age	0.047	1.048	1.001	1.097	0.045^*^
SAS	0.086	1.090	1.015	1.170	0.018^*^
*Parameters at 3700* *m*					
Age	0.054	1.055	1.002	1.111	0.040^*^
Reduction in physical work capacity	0.653	1.922	1.277	2.892	0.002^**^
Fatigues	0.766	2.151	1.412	3.277	<0.001^**^
SAS	0.147	1.158	1.098	1.221	<0.001^**^
ESS	0.060	1.062	1.003	1.126	0.040^*^
*Changes from sea level to 3700* *m*					
Age	0.067	1.070	1.020	1.122	0.005^**^
HR	0.016	1.016	1.003	1.030	0.016^*^
SAS	0.122	1.130	1.086	1.175	<0.001^**^

Age and baseline SAS were independent predictors for sleep disturbances after acute high-altitude exposure

*p < 0.05; **p < 0.01

## Discussion

It is a novel study focusing on the predictive roles of state anxiety for high-altitude induced sleep disturbances in a large sample size of populations (cohort study) who transported acutely to 3700 m by airplane. We found that both the incidence of insomnia and scores for sleep disturbances increased significantly after acute high-altitude exposure. These measurements gradually recovered with exposure time (72 and 168 h), but they remained significantly higher than those at sea level. Associations between sleep disturbances and anxiety, fatigue and other parameters were also observed. Furthermore, predictors of sleep disturbances, including age and SAS score, have been identified. However, in our study, the quality of sleep was not assessed by polysomnography, which may itself disturb sleep; thus, we measured the quality of sleep by multiple dimensionality scales, including AIS, ESS, and self-reported sleep patterns.

### The epidemiology of sleep disturbances and the profiles of SAS, ESS, AIS, and FSAS scores after high-altitude exposure

Sleep disturbances are frequently prevalent in adults and are affected by various factors, including lifestyle, dietary habits, emotional status, and environments^18^. The hypobaric hypoxic environments may be a great negative impact for an individual’s sleep quality, especially after acute exposure without sufficient acclimatization. Sleep disturbances, including insomnia and daytime sleepiness, were significantly lower at sea level before the high-altitude exposure. Upon acute high-altitude stress, sleep disturbances increased markedly and then gradually decreased with exposure time. However, the incidence of sleep disturbances remained higher than that at sea level. This finding is consistent with previous studies that found that individuals at high altitude experienced poor sleep quality and increased sleep disturbances, as assessed by both objective measurements and subjective self-reported questionnaires^3,19^. Sleep quality, as reflected by ESS and AIS scores, was altered in the same pattern as sleep disturbances. High-altitude hypobaric hypoxia is a direct stress on sleep. In addition, poor sleep quality is usually connected to fatigue and anxiety as indicated previously. The other symptoms of AMS may affect insomnia while the insomnia may also influence AMS including its symptoms. In accordance with previous studies, our questionnaires also showed that individuals’ fatigue and anxiety exhibited a similar pattern as sleep disturbances.

The previous has indicated that the Stage 1 nonrapid eye movement sleep (REM) increased while low-wave sleep (SWS) decreased as altitude increased. However, the REM sleep time unchanged^20^. There were various studies that focused on the sleep architecture by using the parameters mentioned above^20^. They are indeed the most important and objective parameters to assess sleep architecture providing direct association of insomnia with that of anxiety state. However, they are recorded by using polysomnography, in a relative small size of population^3,12,20^. However, it is hard to performed polysomnography in a larger size of population, which is a limitation in our study. Thus, we performed three dimensions of scores to assess the high-altitude induced sleep disturbances patterns (self-reported insomnia, AIS score, and ESS score system). We have plan to perform the polysomnography examination to identify the associations between high-altitude induced sleep disturbances and state anxiety as well as depression in the future field study at high altitude.

The modifications of sleep quality, anxiety, and fatigue were partially in accordance with previous reports. The hypobaric hypoxia and cold at high altitude also induce psychological concerns, periodic respiratory distress, fatigue or physical decline, and insomnia, all of which may interact with each other.

### Sleep disturbances are associated with age, HR, and SpO_2_

In the demographic data, only age significantly differed between the groups with and without sleep disturbances, consistent with reported results that older individuals show more sleep issues or insomnia. In the adjusted regression, age remained an independent predictor for sleep disturbances after high-altitude exposure.

High altitude hypobaric hypoxia is a direct stress for sleep. Hypoxia induced the periodic respiration and hyperventilation, activated the state anxiety may be one cause of sleep disturbances. Furthermore, hypocapnia caused by hypoxia may induce sleep disturbances at high altitude^12,16^.

Although HR and SpO_2_ did not significantly differ between the sleep-disturbance and non-sleep-disturbance groups, these parameters were closely related to the AIS score. It is reported that the high-altitude stress mainly due to the hypobaric hypoxia. Regarding to the sleep disturbances, some previous studies have indicated the hypoxia induced hypoxic ventilation reaction play key roles in AMS including insomnia. The high-altitude induced sleep disturbances may associate with hypoxia especially monitored by polysomnography. Furthermore, as your suggestion, we included one index of the hypoxemia, SpO_2_. However, we have not observed the difference of SpO_2_ between sleep disturbances group and non-sleep disturbances group. Neither, association between sleep disturbances and SpO_2_ has not been identified (Tables 1 and 3). However, it is correlated with AIS score. Otherwise, the association between hypoxia and sleep disorders may occur in patients suffered from Sleep Apnea Hypopnea Syndrome, which often caused severe hypoxemia. Furthermore, the arterial blood gases analysis may be the best way to assess the roles of hypoxia on diseases including high-altitude induced sleep disturbances. However, it is an invasive examination that is not suitable to perform at high altitude. However, after adjustment for age and SAS score, they were not independent predictors of sleep disturbances. Regarding the changes in physiological parameters, the change in HR from baseline to high altitude was associated with and a predictor for sleep disturbances. This finding may be because activation of the autonomic nervous system is related to HR variability^21^. Furthermore, a higher HR in individuals exposed to high altitude may be an indicator of high activation of the sympathetic tone, which reflects arousal, especially in the acute or subacute phase.

### The associations between sleep disturbances and state anxiety

It has been demonstrated that insomnia is accompanied by anxiety disorders in those who live at lower altitudes^13^. Many sleep-related problems affect mentally illnesses or anxious patients^22,23^. We found that the incidence of insomnia and sleep disturbances were closely related to the SAS score, although the number of individuals diagnosed with a specific anxiety disorder was insufficient to statistically evaluate the correlation^24,25^. The extent of anxiety at baseline was positively associated with the AIS score at 3700 m, indicating that anxiety plays a causal role in sleep disturbances. Specifically, state anxiety may exacerbate poor sleep quality.

Because sleep is a continuous process that is easily disorganized and interrupted, it is often affected by poor mental or psychological status^25^. However, the SAS score at high altitude was more closely associated with sleep disturbances after 24 h of high-altitude exposure than other factors. It is possible that a high SAS score at baseline causes sleep disturbances after acute high-altitude stress; consequently, poor sleep quality may also increase the anxiety of individuals.

Most of the subjects’ SAS scores increased significantly, but a few of the participants showed a decrease in SAS score. In fact, a large proportion of the participants showed no change in SAS score. It is interesting that the SAS unchanged group exhibited the lowest baseline incidences of insomnia/sleep disturbances and sleepiness as well as the lowest ESS and AIS scores both at baseline and at high altitude. This indicated that stable psychological status may decrease the incidence of sleep disturbances at high altitudes. Furthermore, the SAS-increased group exhibited lower incidences of insomnia, sleep disturbance, and sleepiness at baseline, while the incidences of insomnia, sleep disturbances, and sleepiness after acute hypoxia exposure at 3700 m in this group were higher than those in the other groups. The AIS and ESS scores exhibited the same pattern as sleep disturbances. These results consistently indicate that a stable mental state is of benefit for high sleep quality or, in other words, that individuals with increased anxiety may suffer from poor sleep quality, severe insomnia, and daytime sleepiness.

### Sleep disturbance is also related to sleepiness, fatigue, and physical activity

Sleep disturbances consist mainly of insomnia and disorders that disturb the quality of sleep. Sleepiness is another symptom and dimension of integrated sleep assessment. Poor nocturnal sleep quality usually results in a propensity for daytime sleepiness, which is relevant to mental health. This finding was confirmed by the current study and others’ previous reports^7,11,26^.

Regarding physical activity, it is well recognized that poor sleep quality or insufficient effective sleep may also reduce physical work capacity or increase fatigue. However, the individuals who reported activity reduction before the exposure also showed an increased likelihood of sleep disturbances at high altitude.

### Risk factors and predictors of sleep disturbances

Sleep is affected by a variety of factors, including the specific hypoxic environments^18,27,28^. Thus, acute high-altitude exposure is a great challenge for individuals from lower altitudes in sleep at high altitude^4^. Sleep disorders or sleep disturbances affect individuals’ daily lives. Identifying its risk factors or predictors is of great importance for tourists or workers who will visit high-altitude areas or plateaus.

Finally, in the adjusted regression model, only age and baseline SAS score were independent risk factors and predictors for the onset of sleep disturbances, indicating a causal role of age and SAS score in sleep disturbances, which has not been widely investigated and reported before.

Furthermore, we attempted to identify additional sensitive indicators of sleep disturbances and found that the change in HR and SAS score are also independent predictors of sleep disturbances. However, their predictive values may not be as high as those of baseline age and SAS score.

### Limitations

Our study was performed in young Chinese men, and this selective dataset may potentially generate a bias regarding age or gender; thus, this issue should be addressed in future studies. Another limitation is that we merely performed field trials with acute high-altitude exposure; basic mechanistic studies have not been carried out. These limitations warrant further, more detailed studies.

## Conclusions

High-altitude exposure with hours to a few days, triggers the onset of dramatic sleep disturbances, which are closely associated with state anxiety. Furthermore, age and baseline state anxiety are independent predictors of sleep disturbances at high altitude.

## Supplementary information

Supplementary Figure S1

Supplementary A representative CRF

Supplementary AIS score test

Supplementary ESS score test

Supplementary FSAS test

Supplementary SAS test

Supplementary Detailed Methods

Supplementary Tables S1 and S2

Supplementary Language Editorial Certificate

Supplementary Graphical Abstract

Supplementary Legends and titles for Supplementary Materials
