# Supplementary figures and images for: The onset of sleep disturbances and their associations with anxiety after acute high-altitude exposure at 3700 m
Source: Transl Psychiatry. 2019 Jul 22;9:175. doi: 10.1038/s41398-019-0510-x (PMC6646382; doi:10.1038/s41398-019-0510-x)

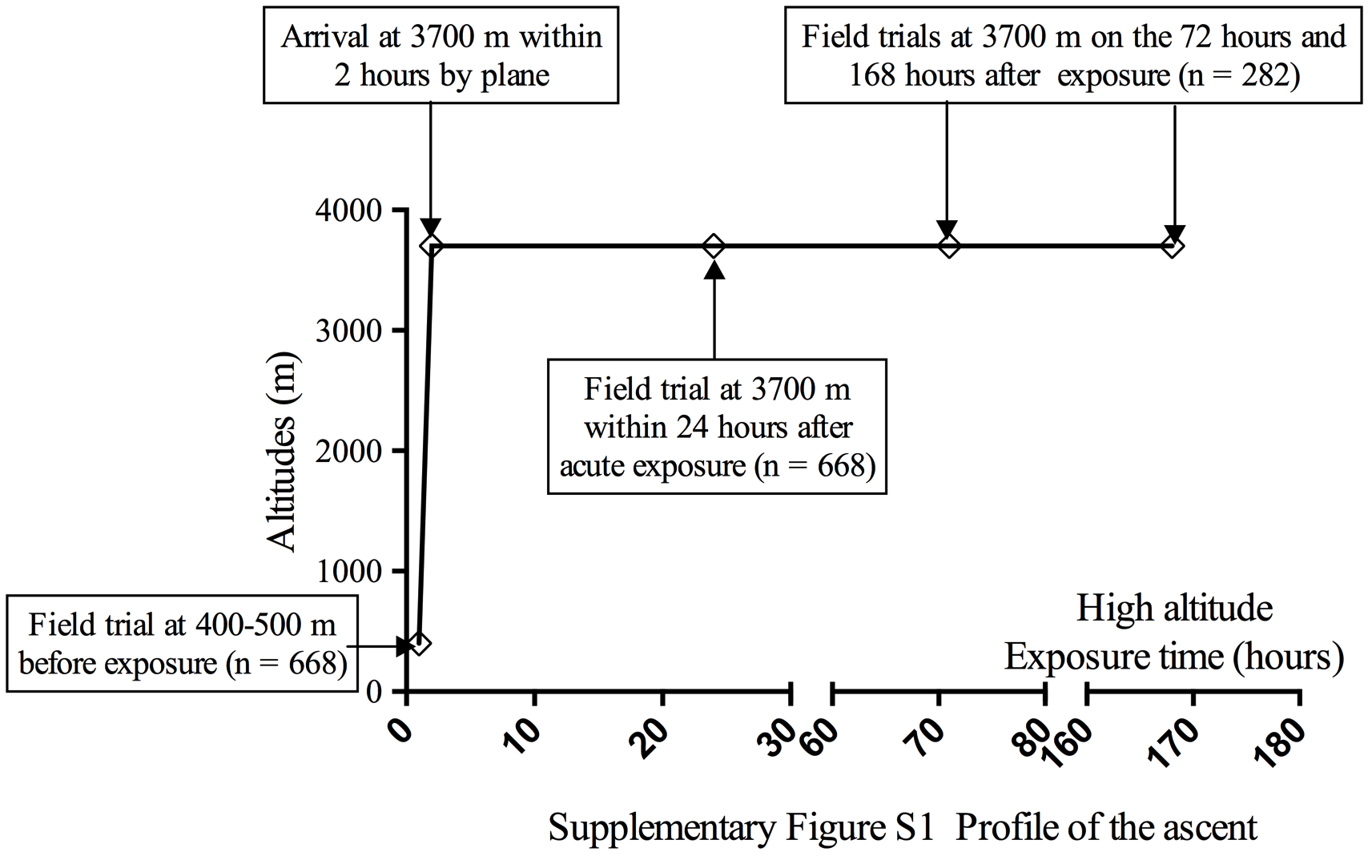

Supplement: Supplementary file 1 — Supplementary Figure S1 [file 41398_2019_510_MOESM1_ESM.tif]
