# Supplementary material for: The onset of sleep disturbances and their associations with anxiety after acute high-altitude exposure at 3700 m
Source: Transl Psychiatry. 2019 Jul 22;9:175. doi: 10.1038/s41398-019-0510-x (PMC6646382; doi:10.1038/s41398-019-0510-x)
Supplement: Supplementary file 3 — Supplementary AIS score test [file 41398_2019_510_MOESM3_ESM.doc]

Athens Insomnia Scale

ID_________ Name_____________ Age__________ Gender_______

**Instructions:** This scale is intended to record your own assessment of any sleep difficulty you might have experienced. Please, check (by circling the appropriate number) the items below to indicate your estimate of any difficulty, provided that it occurred at least three times per week during the last month.

***NO1.*** Sleep induction (time it takes you to fall asleep after turning-off the lights)

0: No problem 1: Slightly delayed

2: Markedly delayed 3: Very delayed or did not sleep at all

***NO2.*** Awakenings during the night

0: No problem 1: Minor problem

2: Considerable problem 3: Serious problem or did not sleep at all

***NO3.*** Final awakening earlier than desired

0: Not earlier 1: A little earlier

2: Markedly earlier 3: Much earlier or did not sleep at all

***NO4.*** Total sleep duration

0: Sufficient 1: Slightly insufficient

2: Markedly insufficient 3: Very insufficient or did not sleep at all

***NO5.*** Overall quality of sleep (no matter how long you slept)

0: Satisfactory 1: Slightly unsatisfactory

2: Markedly unsatisfactory 3: Very unsatisfactory or did not sleep at all

***NO6.*** Sense of well-being during the day

0: Normal 1: Slightly decreased

2: Markedly decreased 3: Very decreased

***NO7.*** Functioning (physical and mental) during the day

0: Normal 1: Slightly decreased

2: Markedly decreased 3: Very decreased

***NO8.*** Sleepiness during the day

0: None 1: Mild

2: Considerable 3: Intense

The period of the self-assessment may vary, depending on the design of a given study. Whenever the self-assessment pertains to a period other than that of the last month, the second sentence of the instructions should be rephrased accordingly.
