# Supplementary material for: The onset of sleep disturbances and their associations with anxiety after acute high-altitude exposure at 3700 m
Source: Transl Psychiatry. 2019 Jul 22;9:175. doi: 10.1038/s41398-019-0510-x (PMC6646382; doi:10.1038/s41398-019-0510-x)
Supplement: Supplementary file 5 — Supplementary FSAS test [file 41398_2019_510_MOESM5_ESM.doc]

**Fatigue Self-Assessment Scale**

ID_________ Name_____________ Age__________ Gender_______

The following 10 statements refer to how you usually feel. For each statement you can choose one out of five answer categories, varying from never to always. 1=never,

2=sometimes; 3=regularly; 4=often; and 5 = always.

|  |  | Never | Sometimes | Regularly | Often | Always |
| --- | --- | --- | --- | --- | --- | --- |
| 1 | I am bothered by fatigue (WHOQOL) | 1 | 2 | 3 | 4 | 5 |
| 2 | I get tired very quickly (CIS) | 1 | 2 | 3 | 4 | 5 |
| 3 | I don’t do much during the day (CIS) | 1 | 2 | 3 | 4 | 5 |
| 4 | I have enough energy for everyday life (WHOQOL) | 1 | 2 | 3 | 4 | 5 |
| 5 | Physically, I feel exhausted (CIS) | 1 | 2 | 3 | 4 | 5 |
| 6 | I have problems starting things (FS) | 1 | 2 | 3 | 4 | 5 |
| 7 | I have problems thinking clearly (FS) | 1 | 2 | 3 | 4 | 5 |
| 8 | I feel no desire to do anything (CIS) | 1 | 2 | 3 | 4 | 5 |
| 9 | Mentally, I feel exhausted | 1 | 2 | 3 | 4 | 5 |
| 10 | When I am doing something, I can concentrate quite well (CIS) | 1 | 2 | 3 | 4 | 5 |
