# Supplementary material for: The onset of sleep disturbances and their associations with anxiety after acute high-altitude exposure at 3700 m
Source: Transl Psychiatry. 2019 Jul 22;9:175. doi: 10.1038/s41398-019-0510-x (PMC6646382; doi:10.1038/s41398-019-0510-x)
Supplement: Supplementary file 6 — Supplementary SAS test [file 41398_2019_510_MOESM6_ESM.doc]

**Self-Rating Anxiety Scale**

How do you feel that which scale is the most appropriate one for you.

1 = none or a little of the time

2 = some of the time

3 = good part of the time

4 = most or all of the time

| Items | Score (1-4) |
| --- | --- |
| 1. I feel more nervous and anxious than usual. |  |
| 2. I feel afraid for no reason at all. |  |
| 3. I get upset easily or feel panicky. |  |
| 4. I feel like I’m falling apart and going to pieces. |  |
| 5. I feel that everything is all right and nothing bad will happen |  |
| 6. My arms and legs shake and tremble. |  |
| 7. I am bothered by headaches neck and back pain. |  |
| 8. I feel weak and get tired easily. |  |
| 9. I feel calm and can sit still easily. |  |
| 10. I can feel my heart beating fast. |  |
| 11. I am bothered by dizzy spells. |  |
| 12. I have fainting spells or feel like it. |  |
| 13. I can breathe in and out easily. |  |
| 14. I get feelings of numbness and tingling in my fingers and toes. |  |
| 15. I am bothered by stomachaches or indigestion. |  |
| 16. I have to empty my bladder often. |  |
| 17. My hands are usually dry and warm. |  |
| 18. My face gets hot and blushes. |  |
| 19. I fall asleep easily and get a good night’s rest. |  |
| 20. I have nightmares. |  |

They were translated into Chinese when used in the trial.
