# Supplementary material for: The onset of sleep disturbances and their associations with anxiety after acute high-altitude exposure at 3700 m
Source: Transl Psychiatry. 2019 Jul 22;9:175. doi: 10.1038/s41398-019-0510-x (PMC6646382; doi:10.1038/s41398-019-0510-x)
Supplement: Supplementary file 7 — Supplementary Detailed Methods [file 41398_2019_510_MOESM7_ESM.docx]

**Supplemental Materials, Methods S1**

**Participants and procedures**

The participants (n = 668) in this study were recruited according to the inclusion and exclusion criteria in June and July of 2012 in Chengdu, Sichuan province (sea level, average 500 m) and Lhasa (3700 m above sea level). Only healthy males between 18 and 40 years of age were included. Subjects with any of the following diseases were excluded: sleep apnea syndrome, hypertension, arrhythmia, myocarditis or other cardiovascular disease, primary headache, cold, pneumonia, pulmonary tuberculosis or other respiratory disease, disorders of the liver or kidneys, malignant tumors and neuropsychosis.

This study was reviewed and approved by the Ethics Committee of Xinqiao Hospital, Army Medical University. The purpose and procedures of the study were thoroughly described to all subjects who agreed to participate, and all of the participants signed informed consent forms before their examinations.

*Procedures and clinical data collection*

Our field trial for the baseline data collection was performed within one week prior to the flight (a two-hour plane ride transported the participants to high altitude on 29 June and 1 July 2012). The trials at high altitude were performed within 18 to 24 hours, at 72 hours (subgroup, n = 282) and at 168 hours (subgroup) after arrival at 3700 m. The trials of this study are shown in figure 1.

Demographic data [*i.e.*, age, body mass index (BMI), smoking history and alcohol consumption] were collected using a structured case report form. Sleep quality was assessed by self-reported sleep patterns (0 = slept as well as usual; 1 = slept poorer than before; 2 = woke up several times; 3 = could not fall asleep last night) and Athens Insomnia Scale (AIS) insomnia scores. Sleepiness was also assessed through self-report (0 = without sleepiness; 1 = with sleepiness) and the Epworth Sleepiness Scale (ESS). Effects on activity were reported as follows: 0 = no activity reduction; 1 = mild activity reduction; 2 = moderate activity reduction; 3 = severe activity reduction. Fatigue was also classified from 0 to 3 depending on the extent of fatigue. Further detailed assessments were measured using a quantitative score (Fatigue Self-Assessment Scale, FSAS). Anxiety was assessed using the widely employed Self-Rating Anxiety Scale (SAS) in Chinese. Systolic blood pressure (SBP), diastolic blood pressure (DBP), heart rate (HR), and pulse oxygen saturation (SpO_2_) were also measured at baseline and at 3700 m after the participants rested for 30 min in a sitting position.

**Definitions of variables**

Sleep disturbances at high altitude were defined as difficulty falling asleep, waking several times or poorer-than-usual sleep quality, or an AIS score greater than 6.

Smoking status was defined as smoking 1 or more cigarettes per day for at least 1 year. Alcohol users were defined as those who drank once a week (clear spirits, beer or red wine).

**Statistical analysis**

Normally distributed variables, including age, BMI, HR, SBP, DBP, and SpO_2_, were expressed as the mean ± SD and were compared using independent sample T tests. Non-normally distributed variables, such as the SAS, AIS, ESS and FSAS scores (including their changes from baseline to high altitude), were expressed as median (interquartile) and compared with non-parametric tests. Furthermore, categorical and non-continuous variables were presented as percentages (cases) and were compared using the Chi square test. The associations between AIS score and other parameters were analyzed by Pearson’s correlation. Univariate and adjusted logistic regressions were used to identify predictors and risk factors for sleep disturbances. A flow chart of the analysis process is provided in figure 2. p ≤ 0.05 was considered statistically significant. The statistical analyses were performed in SPSS 19.0 for Windows. All statistical methods and results were reviewed and approved by statisticians from Army Military Medical University.
