# Supplementary material for: The onset of sleep disturbances and their associations with anxiety after acute high-altitude exposure at 3700 m
Source: Transl Psychiatry. 2019 Jul 22;9:175. doi: 10.1038/s41398-019-0510-x (PMC6646382; doi:10.1038/s41398-019-0510-x)
Supplement: Supplementary file 8 — Supplementary Tables S1 and S2 [file 41398_2019_510_MOESM8_ESM.docx]

Supplementary Tables

Supplementary Table S1 Relationship between AIS and other variables

|  | r | p |
| --- | --- | --- |
| ***Demographic data*** |  |  |
| Age | 0.072 | 0.063 |
| BMI | 0.009 | 0.823 |
| ***Parameters at sea level*** | | |
| Fatigues | 0.248 | <0.001^**^ |
| Reduction in physical work capacity | 0.150 | <0.001^**^ |
| SAS | 0.304 | <0.001^**^ |
| ESS | 0.210 | <0.001^**^ |
| AIS | 0.378 | <0.001^**^ |
| FSAS | 0.265 | <0.001^**^ |
| SBP | -0.003 | 0.937 |
| DBP | 0.021 | 0.594 |
| HR | -0.021 | 0.593 |
| SpO_2_ | -0.025 | 0.521 |
| ***At 3700 m after acute exposure*** | | |
| Fatigues | 0.484 | <0.001^**^ |
| Reduction in physical work capacity | 0.444 | <0.001^**^ |
| SAS | 0.639 | <0.001^**^ |
| ESS | 0.424 | <0.001^**^ |
| FSAS | 0.580 | <0.001^**^ |
| SBP | 0.024 | 0.539 |
| DBP | 0.019 | 0.626 |
| HR | 0.083 | 0.033^*^ |
| SpO_2_ | -0.088 | 0.023^*^ |
| ***Changes from sea level to high altitude*** | | |
| SAS | 0.489 | <0.001^**^ |
| ESS | 0.276 | <0.001^**^ |
| AIS | 0.713 | <0.001^**^ |
| FSAS | 0.37 | <0.001^**^ |
| SBP | 0.023 | 0.556 |
| DBP | 0.013 | 0.734 |
| HR | 0.105 | 0.007^**^ |
| SpO_2_ | -0.089 | 0.021^*^ |

*p < 0.05, ** p < 0.01.

Supplementary Table S2 Univariate logistic regression for sleep disturbances

|  | β | OR | 95%CI | 95%CI | p |
| --- | --- | --- | --- | --- | --- |
| ***Parameters at sea level*** | | | | | |
| Age | 0.057 | 1.059 | 1.013 | 1.108 | 0.018^*^ |
| BMI | 0.013 | 1.013 | 0.937 | 1.094 | 0.749 |
| smoking | -0.198 | 0.821 | 0.595 | 1.132 | 0.228 |
| Drinking | -0.119 | 0.888 | 0.426 | 1.850 | 0.751 |
| History of high altitude exposure | 0.149 | 1.161 | 0.814 | 1.654 | 0.410 |
| Insomnia | 0.722 | 2.058 | 1.217 | 3.478 | 0.007^**^ |
| Sleepiness | 0.583 | 1.792 | 1.065 | 3.014 | 0.028^*^ |
| Fatigues | 0.787 | 2.196 | 1.513 | 3.189 | <0.001^**^ |
| Reduction in physical work capacity | 0.687 | 1.987 | 1.093 | 3.614 | 0.024^*^ |
| SAS | 0.143 | 1.154 | 1.087 | 1.225 | <0.001^**^ |
| ESS | 0.087 | 1.091 | 1.035 | 1.150 | 0.001^*^ |
| AIS | 0.167 | 1.182 | 1.105 | 1.266 | <0.001^**^ |
| FSAS | 0.040 | 1.041 | 1.021 | 1.061 | <0.001^**^ |
| SBP | 0.008 | 1.008 | 0.993 | 1.023 | 0.292 |
| DBP | 0.010 | 1.010 | 0.993 | 1.028 | 0.242 |
| HR | -0.007 | 0.993 | 0.978 | 1.009 | 0.374 |
| SpO_2_ | -0.069 | 0.933 | 0.796 | 1.095 | 0.396 |
| ***Parameters at 3700 m*** | | | | | |
| Sleepiness | 0.985 | 2.679 | 1.774 | 4.045 | <0.001^**^ |
| Fatigues | 1.607 | 4.986 | 3.517 | 7.067 | <0.001^**^ |
| Reduction in physical work capacity | 1.420 | 4.138 | 2.950 | 5.804 | <0.001^**^ |
| SAS | 0.211 | 1.235 | 1.175 | 1.298 | <0.001^**^ |
| ESS | 0.153 | 1.165 | 1.109 | 1.224 | <0.001^**^ |
| FSAS | 0.067 | 1.070 | 1.050 | 1.090 | <0.001^**^ |
| SBP | 0.002 | 1.002 | 0.989 | 1.016 | 0.743 |
| DBP | 0.003 | 1.003 | 0.987 | 1.019 | 0.721 |
| HR | 0.013 | 1.013 | 0.999 | 1.026 | 0.063 |
| SpO_2_ | -0.039 | 0.961 | 0.913 | 1.013 | 0.137 |
| ***Changes from sea level to 3700 m*** | | | | | |
| SAS | 0.122 | 1.129 | 1.087 | 1.174 | <0.001^**^ |
| ESS | 0.078 | 1.081 | 1.034 | 1.130 | 0.001^**^ |
| AIS | 0.349 | 1.417 | 1.322 | 1.520 | 0.001^**^ |
| FSAS | 0.033 | 1.033 | 1.018 | 1.048 | <0.001^**^ |
| SBP | -0.003 | 0.997 | 0.986 | 1.008 | 0.577 |
| DBP | -0.005 | 0.995 | 0.982 | 1.009 | 0.522 |
| HR | 0.016 | 1.016 | 1.003 | 1.029 | 0.013^*^ |
| SpO_2_ | -0.030 | 0.970 | 0.923 | 1.020 | 0.239 |

*p < 0.05, ** p < 0.01.
