# Supplementary material for: The onset of sleep disturbances and their associations with anxiety after acute high-altitude exposure at 3700 m
Source: Transl Psychiatry. 2019 Jul 22;9:175. doi: 10.1038/s41398-019-0510-x (PMC6646382; doi:10.1038/s41398-019-0510-x)
Supplement: Supplementary file 10 — Supplementary Graphical Abstract [file 41398_2019_510_MOESM10_ESM.pptx]

## Slide 1
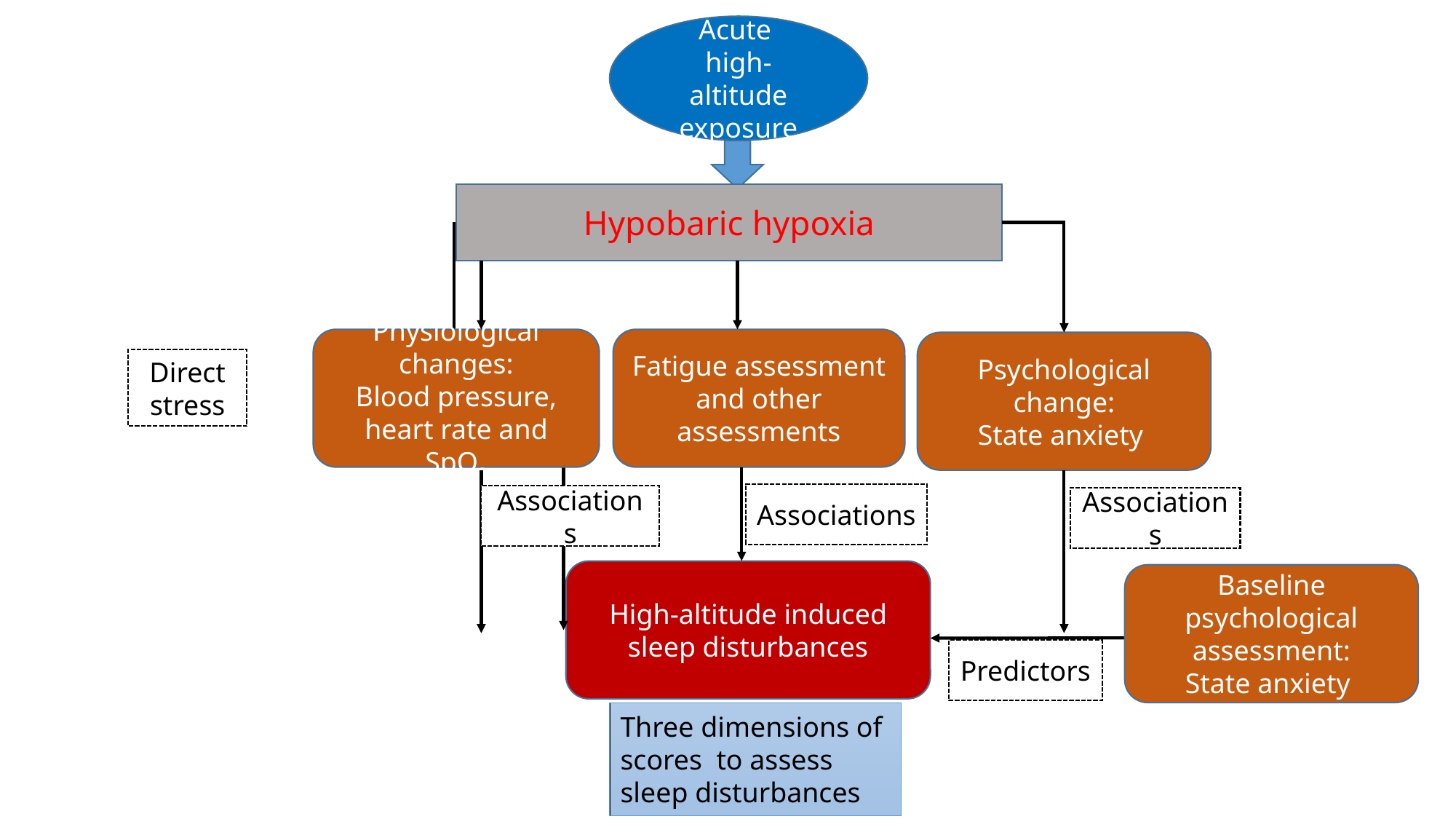

Acute
high-altitude exposure
Hypobaric hypoxia
Fatigue assessment
and other assessments
Physiological changes:
Blood pressure, heart rate and SpO2
Psychological change:
 State anxiety
Direct stress
Associations
Associations
Associations
High-altitude induced sleep disturbances
Baseline psychological assessment:
 State anxiety
Predictors
Three dimensions of scores to assess sleep disturbances
