# Supplementary material for: The onset of sleep disturbances and their associations with anxiety after acute high-altitude exposure at 3700 m
Source: Transl Psychiatry. 2019 Jul 22;9:175. doi: 10.1038/s41398-019-0510-x (PMC6646382; doi:10.1038/s41398-019-0510-x)
Supplement: Supplementary file 11 — Supplementary Legends and titles for Supplementary Materials [file 41398_2019_510_MOESM11_ESM.docx]

**Legends for Supplementary Materials**

1. Supplementary Material 1 Supplementary Figure S1: The profiles of ascent to high altitude

The subjects were transported to 3700 m by airplane in two hours, and the examinations were performed at 24, 72 and 168 hours after exposure at high altitude.

2. Supplementary Material 2 a representative CRF

3. Supplementary Material 3 AIS score test

4. Supplementary Material 4 ESS score test

5. Supplementary Material 5 FSAS test

6. Supplementary Material 6 SAS test

7. Supplementary Material 7 Detailed Methods

8. Supplementary Material 8 Supplementary Tables S1 and S2

Supplementary Table S1 Relationship between AIS and other variables

Supplementary Table S2 Univariate logistic regression for sleep disturbances

9. Supplementary Material 9 Language Editorial Certificate

10. Supplementary Material10 Graphical Abstract

11. Supplementary Material 11 Legends and titles for Supplementary Materials
